# Known disease‐causing variants in homologous proteins help predict pathogenicity of SORL1 variants in Alzheimer’s disease

**DOI:** 10.1002/alz.084835

**Published:** 2025-01-03

**Authors:** Olav M. Andersen, Giulia Monti, Anne Mette Gissel Jensen, Matthijs W J de Waal, Marc Hulsman, Johan G Olsen, Henne Holstege

**Affiliations:** ^1^ Aarhus University, Aarhus C Denmark; ^2^ Aarhus University, Aarhus Denmark; ^3^ Amsterdam UMC, Amsterdam Netherlands; ^4^ Copenhagen University, Copenhagen, Copenhagen Denmark; ^5^ Amsterdam UMC location VUmc, Amsterdam, Noord‐holland Netherlands

## Abstract

**Background:**

*SORL1* encodes the retromer‐associated receptor SORLA that functions in endosomal recycling. Rare variants in *SORL1* have been associated with Alzheimer’s disease (AD) and rare pathogenic variants are estimated to occur in up to 2.75% of early onset AD patients and in 1.5% of unrelated late onset AD patients. While truncation mutations are observed almost exclusively in AD patients, it is currently unknown which among the hundreds of rare missense variants identified in *SORL1*, are pathogenic.

**Method:**

Here we address this question by relying on SORLA’s distinct molecular architecture. First, we completed a structure‐guided sequence alignment for all the protein domains. Next, we identified proteins that contain domains homologous to those of SORLA, which include pathogenic variants for monogenic diseases.

**Result:**

We identified the analogous domain positions of these variants in the SORLA protein sequence and showed that variants in these positions similarly impair *SORL1*, and lead to AD. Together, our findings represent a comprehensive study on SORLA protein variation and functional effects, which allowed us to prioritize *SORL1* genetic variants into high or moderate priority mutations.

**Conclusion:**

We envision that our findings will be used by clinical geneticists for assessing variants they identify in patients, allowing further development of diagnostic procedures and patient counseling strategies. Ultimately, the identification of the most pathogenic variants will inform investigations into the molecular mechanisms of endosomal recycling which will support the development of therapeutic treatment strategies for *SORL1* variant‐carrying patients.

